# Naringin Decreases TNF-α and HMGB1 Release from LPS-Stimulated Macrophages and Improves Survival in a CLP-Induced Sepsis Mice

**DOI:** 10.1371/journal.pone.0164186

**Published:** 2016-10-07

**Authors:** Minchan Gil, Yun Kyu Kim, Sang Bum Hong, Kyung Jin Lee

**Affiliations:** 1 Nano-Bio Resources center, Department of Cosmetic Sciences, Sookmyung Women's University, Seoul, Republic of Korea; 2 Department of Convergence Medicine, Asan Institute for Life Sciences, University of Ulsan College of Medicine, Asan Medical Center, Seoul 05505, Republic of Korea; 3 Division of Pulmonary and Critical Care Medicine, Department of Internal Medicine, Asan Medical Center, University of Ulsan College of Medicine, Seoul 05505, Republic of Korea; National Institutes of Health, UNITED STATES

## Abstract

Naringin, a flavanone glycoside extracted from various plants, has a wide range of pharmacological effects. In the present study, we investigated naringin’s mechanism of action and its inhibitory effect on lipopolysaccharide-induced tumor necrosis factor-alpha and high-mobility group box 1 expression in macrophages, and on death in a cecal ligation and puncture induced mouse model of sepsis. Naringin increased heme oxygenase 1 expression in peritoneal macrophage cells through the activation of adenosine monophosphate-activated protein kinase, p38, and NF-E2-related factor 2. Inhibition of heme oxygenase 1 abrogated the naringin’s inhibitory effect on high-mobility group box 1 expression and NF-kB activation in lipopolysaccharide-stimulated macrophages. Moreover, mice pretreated with naringin (200 mg/kg) exhibited decreased sepsis-induced mortality and lung injury, and alleviated lung pathological changes. However, the naringin’s protective effects on sepsis-induced lung injury were eliminated by zinc protoporphyrin, a heme oxygenase 1 competitive inhibitor. These results revealed the mechanism underlying naringin’s protective effect in inflammation and may be beneficial for the treatment of sepsis.

## Introduction

Sepsis caused by aggressive infection is a significant public healthcare problem with an increasing incidence that affects millions of people worldwide each year; one of every four people affected is killed [1]. Septic myocardial dysfunction is thought to be regulated by various cytokines released from innate immune cells, including tumor necrosis factor (TNF)- α, interleukin-1, interferon-r, and high-mobility group box 1 (HMGB1) [2–4]. Among the cytokines involved in sepsis, TNF-α, a primary mediator of septic shock, is secreted immediately after infection and triggers the pathological process of septic shock. On the other hand, HMGB1 has been identified as a late mediator of sepsis [4] and plays a critical role in endothelial cell barrier disruption by rearranging the actin cytoskeleton into a contractile phenotype [5]. It contributes to the high lethality of sepsis via late-acting downstream effectors [6,7]. Interestingly, HMGB1 inhibitors and neutralizing antibodies significantly increase survival in septic patients, suggesting that HMGB1 might be a therapeutic target for sepsis [8].

The stress responsive heme oxygenase (HO-1) 1 decreases serum HMGB1 levels in animal models of sepsis and improves patient survival, thereby demonstrating its therapeutic potential in sepsis [8,9]. Our previous study also showed that HO-1 can improve survival and decrease serum HMGB1 levels in a mouse model of sepsis, further indicating the beneficial effects of HO-1 in inflammatory disorders [10]. Indeed, many kinase signaling pathways, including adenosine monophosphate-activated protein kinase (AMPK) and p38 mitogen-activated protein kinase (MAPK), are involved in HO-1 gene expression. Moreover, previous studies provided evidence that AMPK and/or p38 MAPK play key roles in the induction of HO-1 in endotoxemia-induced lung injury [8,11]. Under oxidative conditions, increased expression of the HO-1 gene is mediated by the transcription factors NF-kB, activating protein 1 (AP-1), and NF-E2-related factor 2 (Nrf2) [12–14]. Therefore, HO-1 and its regulatory pathway might provide targets for therapeutic intervention for inflammatory disorders like sepsis.

Naringin, a flavanone glycoside, is widely distributed in various plants and is an important component of the herb *Drynaria*. Naringin has a wide range of biological properties, including anti-inflammatory, antioxidant, antibacterial, and anti-apoptotic activities [15]. Specifically, naringin inhibits lipopolysaccharide (LPS)-induced production of nitric oxide by suppressing the activation of nuclear factor kappa-light-chain-enhancer of activated B cells (NFkB) in RAW 264.7 cells [16,17]. Moreover, naringin ameliorates LPS-induced acute lung injury, mainly by reducing neutrophil migration into the lungs, which attenuates pro-inflammatory cytokine production and subsequent lung disease [18]. However, the role of naringin in HMGB1 release and the related molecular mechanisms remain to be elucidated.

The present study investigated the role of naringin in the release of the inflammatory cytokines TNF-α and HMGB1 from macrophages. Our findings suggest that an AMPK–p38–Nrf-2-mediated increase in HO-1 is the key molecular pathway involved in naringin’s anti-inflammatory effect.

## Materials and Methods

### Cells, Antibodies, and Reagents

RAW 264.7 cells were cultured in Rosewell Park Memorial Institute-1640 Medium supplemented with 10% fetal bovine serum, penicillin, and 100 μg/ml streptomycin (Gibco BRL Gaithersburg, MD). Antibodies against AMPK, p-AMPK, acetyl-CoA carboxylase (ACC), p-ACC, p38, and phospho-p38 (p-p38) were purchased from Cell Signaling Technology (Beverly, MA). Anti-HMGB1 antibody was purchased from Abcam (Cambridge, MA). Anti-HO-1 antibody, anti-β-actin antibody, scrambled siRNAs, and siAMPKα1 were obtained from Santa Cruz Biotechnology (Santa Cruz, CA). The enhanced chemiluminescence western blotting detection reagent was purchased from Amersham (Buckinghamshire, UK). The p38 MAPK inhibitors (PD98059, SB203580, and SP600125) were purchased from Calbiochem (San Diego, CA). All other chemicals, including LPS (*Escherichia coli* 0111:B4), naringin, zinc protoporphyrin (ZnPP; an inhibitor of HO-1 enzyme), and MTT (3-[4,5-dimethylthiazol-2-yl]-2,5-diphenyltetrazolium bromide) were purchased from Sigma-Aldrich (St. Louis, MO). LPS and inhibitors were treated on cultured cells with appropriate concentration: 1 μg/ml of LPS, 10 μM of PD989052, SB203580, SP600125, and ZnPP.

### Animals

Specific pathogen-free 8 weeks-old BALB/C male mice (weight 20–22g) were purchased from Central Laboratory Animal Inc. (Seoul, Republic of Korea). Mice were housed and maintained in a controlled specific pathogen-free conditions at 21–24°C and 40–60% relative humidity under a 12-h light/dark cycle with free access to food and water. animals were provided with veterinary/supportive care when they began to show signs of illness. All animal experiments were performed in accordance with the Korean Ministry of Food and Drug Safety (MFDS) guidelines.

### Animals Ethics statement

All protocols were reviewed and approved by the Institutional Animal Care and Use Committees (IACUC) of Asan Institute for Life Sciences (Permit Number: 2015-12-119). All mice were maintained in the specific pathogen–free facility of the Laboratory of Animal Research in the Asan Medical Center (AMClar). All mice were handled with regard for alleviation of suffering. Analgesics were not used as treatment in mice after sepsis induction, due to their possible interference via the production of inflammatory mediators. After sepsis induction, mice showed piloerection, crusty exudates around their eyes, reduced locomotion and altered breath frequency. These events were worsened as the mice approached death. Most animals were euthanized in 30 min after we found the severe symptoms by overdose of ketamine and xylazine (>100/10 mg/kg, s.c.) followed by cervical dislocation. Total number of euthanized mice were 15.

For survival experiments, we monitored mice with CLP-induced sepsis at each 12 h for 8 days. At this time, mice that show signs of imminent death were euthanized by ketamine/xylazine overdose followed by cervical dislocation. At the end of the survival experiment, live mice were also euthanized by ketamine/xylazine overdose followed by cervical dislocation.

### Viability assay

Naringin was dissolved in dimethyl sulfoxide (DMSO) and added directly to the culture media. The final concentration of DMSO was always <0.1%. Each well of a 48-well plate was seeded with 5 × 10^3^ cells, which were incubated for 24 h. Various concentrations of naringin and LPS (1μg/ml) were added to the well, and the plates were incubated at 37°C for an additional 24 h. After the supernatant was removed, the cells were used in the MTT assay. Relative cytotoxicity was quantified by absorption measurements at 550 nm using a microtiter plate reader (Molecular Devices, Menlo Park, CA). This wavelength was not found to interfere with naringin.

### Enzyme-linked immunosorbent assays

TNF-α and HMGB1 levels in culture medium and blood were determined using enzyme-linked immunosorbent assay (ELISA) kits (R&D Systems) according to the manufacturer’s instructions. Briefly, polyclonal rat anti-mouse cytokine antibodies were used as primary antibodies and biotinylated polyclonal rat anti-mouse cytokine antibodies were used for detection; a standard curve was generated for each assay. Color changes were determined at 450 nm.

### RNA preparation and real-time quantitative PCR

Total RNA was extracted from cells using Trizol (Gibco BRL) and reverse-transcribed using the iScript cDNA synthesis kit (Bio-Rad Laboratories, Hercules, CA). The cDNA was amplified using specific primers for HO-1, TNF-α, and HMGB1. Real-time polymerase chain reaction (RT-PCR) was performed using an iCycler iQ system with the iQ SYBR Green Supermix (Bio-Rad Laboratories). The quantity of each transcript was calculated with the comparative Ct method and normalized using the housekeeping gene S18.

The following primer sequences were used: the HO-1 primers were sense 5′-CGCCTTCCTG CTCAACATT-3′ and antisense 5′-TGTGTTCCTC TGTCAGCATC AC-3′; the HMGB1 primers were sense 5′-TTGTGCAAAC TTGCCGGGAG GA-3′ and antisense 5′-ACTTCTCCTTCAGCTTGGCAGC-3′; the TNF-α primers were sense 5′-AGCCCACGTCGTAGCAAACCACCAA-3′ and antisense 5′-AACACCCATT CCCTTCAC -AGAGCAAT-3′; and the mouse ribosomal protein S18 primers were sense 5′- AGTTCCAGCACATTTTGCGAG-3′ and antisense 5′-TCATCCTCCGTGAGTTCTCCA-3′. The quantity of each transcript was calculated as described in the instrument manual and normalized using the housekeeping gene S18.

### Western blot analysis

Protein samples were heated at 95°C for 5 min, analyzed using sodium dodecyl sulfate polyacrylamide gel electrophoresis (SDS-PAGE) and electrophoretically transferred to an Immune-Blot™ polyvinylidene difluoride (PVDF) membrane (Bio-Rad Laboratories). The membranes were incubated with primary rabbit antibodies, which included anti-TNF-α, anti-HO-1, anti-HMGB1, anti-AMPK, anti-phospho-AMPK, anti-ACC, anti-phospho-ACC, anti-p38, anti-p-p38, anti-Nrf2, anti-NF-kB p65, and anti-β-actin antibodies. Immunoblot signals were developed by enhanced chemiluminescence (Pierce Biotechnology, Rockford, IL), analyzed by the ImageQuant™ LAS 4000 biomolecular imager (GE Healthcare Life Sciences, Waukesha, WI) and bundled Multi Gauge 3.0 software. Values of western blot in figures represent relative density of the bands normalized to that of β-actin.

### SiRNA knockdown

SiRNA transfection into macrophages was performed using the transfection reagent Lipofectamine® 2000 (Invitrogen, Carlsbad, CA) according to the manufacturer’s instructions. The cells were transfected with 100 nM of target siRNA or scrambled siRNA. The transfected cells were then incubated for 18 h in medium. The cells were then washed, pretreated with or without naringin for 1 h, and treated with LPS.

### Transient transfection and luciferase assay

3 × 10^5^ cells were seeded in each well of 24-well plates, incubated overnight, and transiently co-transfected with antioxidant response element (ARE) or a NF-**κ**B-promoter-luciferase construct and pRL-SV40 plasmid (Renilla luciferase expression for normalization) (Promega, Madison, WI) using Lipofectamine® 2000 reagent. Relative luciferase activities were generated by normalizing promoter-driven firefly luciferase activity with the level of Renilla luciferase activity.

### Animal model of sepsis

For the cecal ligation and puncture (CLP)-induced sepsis experiments, BALB/c mice (male, 8 weeks old, 20–25 g) were anesthetized with ketamine (30 mg/kg) and xylazine (6 mg/kg). Next, a 2-cm midline incision was made to expose the cecum and the adjoining intestines. The cecum was tightly ligated with a 3.0 silk suture at 5.0 mm from the cecal tip and perforated once with a 22-gauge needle. The cecum was then gently squeezed to extrude a small amount of feces to ensure patency of the hole. The bowel was then returned to the abdomen and the incision was closed with a 4.0 silk suture. For sham animals (n = 10), the cecum was exposed, but not ligated or punctured, and returned to the abdomen. To evaluate the naringin’s effect on the survival of CLP mice, mice were treated with either vehicle (DMSO, 0.1 ml per mouse, n = 5), naringin (10 mg/kg, intraperitoneal, n = 10), or naringin (10 mg/kg) with ZnPP (10 mg/kg, intraperitoneal, n = 10) 2 h prior to and 12 h after the operation. Survival was monitored every 24 h for up to 8 days.

### Organ injury experiments

The superior lobe of the right lung was excised and fixed with 4% paraformaldehyde in phosphate-buffered saline. Fixed tissues were embedded in paraffin wax, sectioned into 4 μm-thick lung sections, and then subjected to hematoxylin and eosin staining for histopathological examination. The lung injury scores were calculated in sections by assessing neutrophil infiltration, hemorrhage, necrosis, congestion, and edema [18]. Each injury was scored according to the following system: 0 = normal; 1 = ≤25%; 2 = 25–50%; 3 = 50–75%; 4 = ≥75%.

### Measurement of serum TNF-α and HMGB1 levels

The CLP operation was performed as described above. The animals were randomly divided into four groups: (1) sham (n = 5); (2) CLP (n = 10); (3) CLP + naringin (1 mg/kg and 10 mg/kg, respectively, intraperitoneal, n = 10); and (4) CLP + naringin (10 mg/kg) + ZnPP (10 mg/kg, intraperitoneal, n = 10). The mice were treated with naringin 2 h prior to and 12 h after the CLP operation. ZnPP was administered 2 h prior to the CLP operation. Twenty-four hours after CLP, all animals were sacrificed under ketamine anesthesia (30 mg/kg, intraperitoneal). Blood samples were obtained and centrifuged using a fixed-angle (35°) centrifuge at 7500 × *g* for 20 min at 4°C. ELISA kits for TNF-α and HMGB1 (Shino-Test Corp., Tokyo, Japan) were used to verify the serum HMGB1 and TNF-α levels in the Sham and CLP-treated animals. All blood and tissue sampling procedures were carried out aseptically in specific pathogen-free conditions facilities.

### Statistical analysis

All experiments were repeated at least three times. The results are expressed as the mean ± standard deviation. The levels of significance for comparison between group differences were compared using a one-way analysis of variance, followed by a Student’s *t*-test. A *P* value < 0.05 was considered statistically significant. The Kaplan–Meier method was used to compare between-group differences in mortality rates.

## Results

### Naringin inhibits the release and expression of cellular TNF-α and HMGB1 in LPS-stimulated macrophages

**Fig 1:**
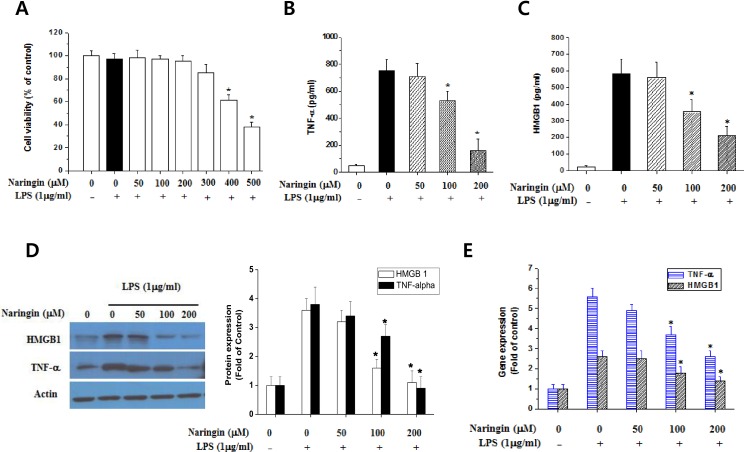
Naringin’s effect on the expression of TNF-α and HMGB1 in LPS-stimulated macrophages. Cells were pretreated with naringin for 1 h at the doses indicated in the figure. The cells were then stimulated with LPS (1 μg/ml) for 24 h. Cell viability was measured by MTT assays (A). The culture medium was collected and subjected to ELISA to measure the TNF-α (B) and HMGB1 (C) concentrations. Cells were harvested, lysed, and subjected to western blot (D) and RT-PCR (E) to determine TNF-α and HMGB1 levels. *Significantly different from LPS-treated cells.

A previous study reported that naringin inhibits the LPS-induced production of inflammatory cytokines in RAW 264.7 cells [16]. Thus, to identify the mechanism underlying naringin’s anti-inflammatory effect, naringin’s effect on peritoneal macrophage cell viability was assessed using an MTT assay. No significant cytotoxicity was observed at naringin concentrations of up to 200 μM (>95% cell viability) (Fig 1A). Then, we measured the expression of the inflammatory cytokines TNF-*α* and HMGB1. Cells were incubated with naringin in the presence of LPS. Naringin repressed TNF-*α* and HMGB1 release from LPS-stimulated macrophages in a concentration-dependent manner (Fig 1B and 1C). Naringin also dose-dependently reduced cellular TNF-α and HMGB1 (Fig 1D) and mRNA (Fig 1E) expression. Thus, we confirmed naringin’s inhibition of TNF-α expression, as reported previously [16], and showed, for the first time, that naringin inhibits HMGB1 release from LPS-stimulated macrophages.

### Naringin induces HO-1 protein and gene expression via the AMPK signaling pathway in macrophages

**Fig 2:**
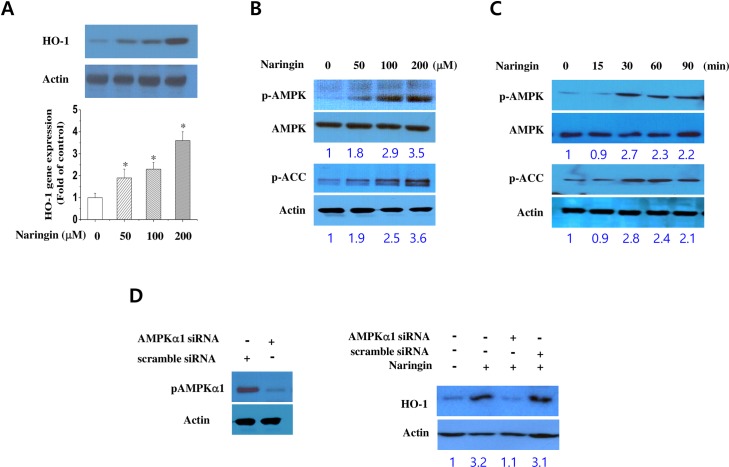
Naringin’s effect on the AMPK-mediated HO-1 expression in macrophages. Cells were exposed to various concentrations of naringin for 8 h. HO-1 mRNA and protein expression were analyzed by RT-PCR and western blotting (A). Phosphorylated AMPK (p-AMPK), AMPK, phosphorylated ACC (p-ACC), and ACC expression were analyzed by western blotting with various concentrations of naringin at 90 min (B) and at 15, 30, 60, and 90 min after treatment with 200 μM naringin (C). Knockdown of AMPKα1 with siRNA treatment was confirmed by western blotting (D). HO-1 expression was measured in AMPKα1-silenced macrophages either with or without naringin treatment. *Significantly different from control.

We then examined whether naringin induces the expression of anti-inflammatory genes like the HO-1 gene. Treating cells with naringin for 24 h dose dependently increased HO-1 mRNA and protein expression (Fig 2A). Previous studies have reported that AMPK signaling controls HO-1 induction in immune disease models [19,20]. Therefore, we wanted to determine if naringin induces HO-1 expression via the activation of AMPK signaling. We found that naringin increased the phosphorylation of AMPK and ACC, a downstream target of AMPK, in a concentration- and time-dependent manner (Fig 2B and 2C). In addition, silencing AMPK reversed naringin’s effect on HO-1 expression (Fig 2D). Therefore, we demonstrated for the first time that activation of AMPK signaling is required for naringin-induced HO-1 expression in macrophages.

### Naringin induces HO-1 protein via the activation of p38 MAPK and Nrf-2 signaling in macrophages

**Fig 3:**
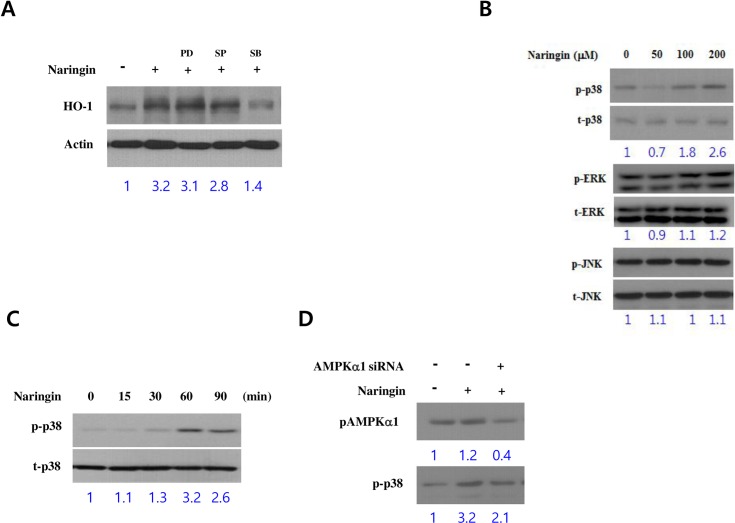
Role of the p38 kinase pathway in naringin-induced HO-1 expression. Cells were pretreated with SB203580 (10 μM), SP600125 (10 μM), or PD98059 (10 μM) for 1 h and then with naringin (200 μM) for 8 h. The cells were then harvested and subjected to western blotting (A). The amount of Phosphorylated p38, JNK, Erk and total kinases was analyzed by various concentrations of naringin for 8 h (B). p-p38 and total p38 kinase (t-p38) expression was analyzed by western blotting at 15, 30, 60, and 90 min after cells were treated with naringin (200 μM) (C). p-p38 and p-AMPKα expression in AMPKα1 knockdown and control cells after naringin treatment were analyzed by western blotting (D).

We previously reported that p38 MAPK activation by a natural compound induces HO-1 expression in macrophages [21]. Therefore, we examined whether p38 MAPK is responsible for naringin-induced HO-1 expression. To identify which MAPK mediates naringin-induced HO-1 expression, pharmacological inhibitors of MAPKs were used. Naringin-induced HO-1 expression in macrophage cells was inhibited by the p38 inhibitor SB203580, but not by the extracellular-signal-regulated kinase (ERK) inhibitor PD98059 or c-Jun N-terminal kinase (JNK) inhibitor SP600125 (Fig 3A). Moreover, naringin induced p38 activation in macrophages in a concentration- and time-dependent manner (Fig 3B and 3C). These results suggested that p38 plays a key role in naringin-induced HO-1 expression. p38 activation was evident 1 h after naringin treatment; this was in contrast to AMPK activation, which appeared 30 min after treatment. We then investigated whether AMPK activation is necessary for p38 activation by silencing AMPK (Fig 3D). Suppressed p38 activation in cells with reduced p-AMPKα1 expression, due to AMPK knockdown, suggested that naringin-induced AMPK activation lies upstream of p38 activation in macrophages.

**Fig 4:**
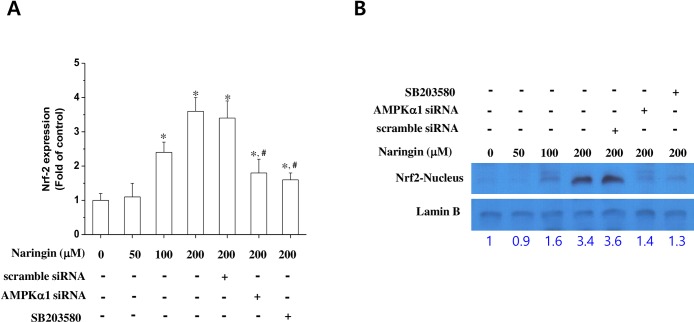
Naringin’s effect on Nrf2 nuclear translocation and transcriptional activity. Macrophages transiently transfected with ARE–luciferase reporter plasmid were treated with naringin at the indicated concentrations with or without AMPKα knockdown by siRNA or SB203508 (10 μM) and subjected to a luciferase assay (A). Macrophages were treated with naringin at the indicated concentrations for 24 h with or without AMPKα knockdown by siRNA or SB203508 (10 μM). Nuclear extracts were then subjected to western blotting to determine Nrf-2 expression levels (B). *Significantly different from control. ^#^Significantly different from cells treated with naringin + LPS.

It has recently been recognized that Nrf2 regulates ARE-driven HO-1 gene expression [13,22]. To assess whether AMPK and p38 MAPK play key roles in Nrf2-induced HO-1 expression, we examined naringin’s effect on ARE–luciferase activity and Nrf2 nuclear translocation. As previously reported [13,22], naringin stimulated ARE–luciferase activity (Fig 4A) and Nrf2 nuclear translocation (Fig 4B) in a concentration-dependent manner. To investigate the relationship between naringin-induced p38 and AMPK activation, we used SB203580, a specific p38 MAPK inhibitor, and AMPK silencing. Naringin-induced ARE–luciferase activity and Nrf-2 nuclear translocation were significantly reduced by both SB203580 and AMPK silencing (Fig 4A and 4B). Overall, these results suggest that naringin induces HO-1 expression in macrophages via the AMPK–p38–Nrf2 pathway.

### Naringin inhibits NF-κB luciferase activity and nuclear translocation

**Fig 5:**
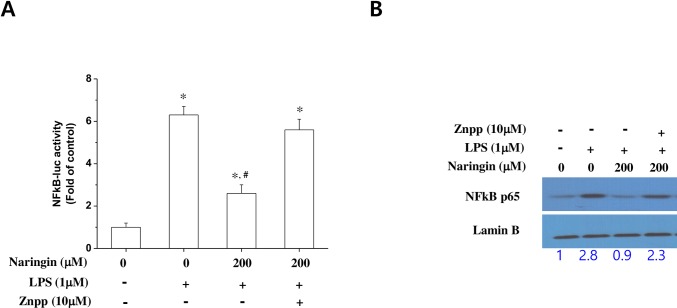
Effect of the HO-1 inhibitor ZnPP on LPS-induced naringin inhibition of NF-κB activation in macrophages. Macrophages transiently transfected with NF-κB–luciferase reporter plasmid were treated with LPS, LPS + naringin, or LPS + naringin + ZnPP for 24 h, and subjected to a luciferase assay (A). Macrophages were treated with LPS, LPS + naringin, or LPS + naringin + ZnPP for 24 h, and nuclear extracts were subjected to western blotting to determine NF-κB subunit p65 expression levels (B). *Significantly different from control. ^#^Significantly different from cells treated with naringin + LPS.

Naringin has been shown to inhibit NF-κB in an LPS-stimulated macrophage cell line and in mice [18,23]. We examined whether naringin inhibits NF-κB-dependent gene expression by increasing HO-1 protein levels, using luciferase reporter assays. Naringin clearly inhibited LPS-stimulated NF-κB luciferase activity. Interestingly, naringin’s effect on NF-κB activity was inhibited by ZnPP, an inhibitor of the HO-1 enzyme (Fig 5A). Western blotting results were consistent with this those of the luciferase reported assay, as they revealed that naringin reduced the nuclear expression levels of the NF-κB subunit p65 in LPS-stimulated macrophages. Naringin-induced NF-κB nuclear translocation was also completely reversed by ZnPP (Fig 5B). These results suggest that naringin suppresses NF-κB activation by increasing HO-1 expression.

### P38, AMPK and HO-1 mediates the LPS-stimulated release of TNF-α and HMGB1 from macrophages

**Fig 6:**
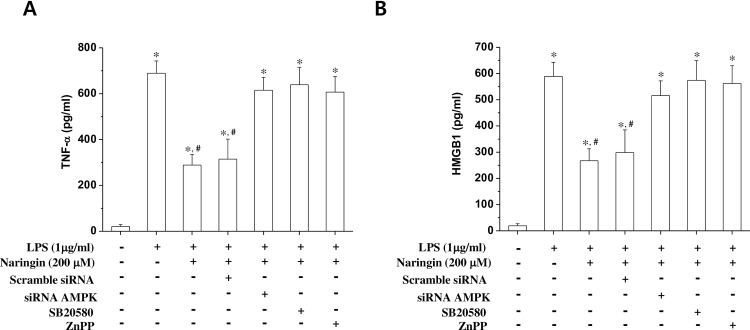
AMPK, p38, and HO-1’s effects on naringin-induced inhibition of LPS-induced TNF-α and HMGB1 release. Cells were transfected with AMPKα1-siRNA or scrambled siRNA, treated with naringin (200 μM) either with or without SB203580 (10 μM) and ZnPP (10 μM) for 1 h, and then stimulated with LPS (1 μg/ml) for 24 h. Culture supernatants were then subjected to ELISA to measure TNF-α (A) and HMGB1 (B) expression levels. *Significantly different from control. ^#^ Significantly different from cells treated with naringin + LPS.

We showed that naringin induces HO-1 protein expression through the activation of the AMPK and p38 signaling pathways. It also remained to be determined whether these naringin-stimulated signaling pathways inhibited TNF-α and HMGB1 release. We used ZnPP, SB203580, and siRNA-AMPK to analyze the effect of the specific inhibition of their respective signaling pathways on the naringin-induced reduction of TNF-α and HMGB1 release from LPS-stimulated macrophages. Our results showed that naringin-induced inhibition of TNF-α (Fig 6A) and HMGB1 (Fig 6B) release was also reversed in the presence of SB203580, siRNA-AMPK, and ZnPP. Thus, we propose that p38, AMPK and HO-1 signaling is an important component of the naringin-induced HO-1 expression’s anti-inflammatory activity.

### Naringin improves survival in the CLP sepsis model via HO-1

**Fig 7:**
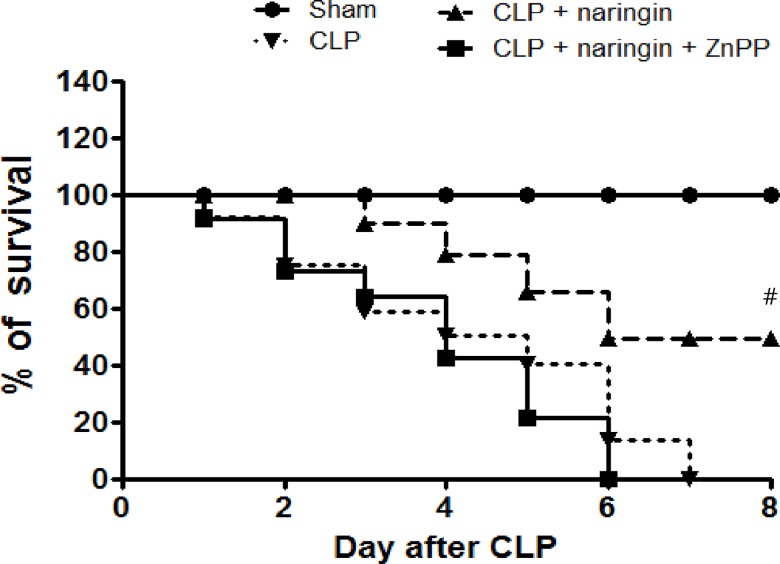
Naringin’s effect on the survival of CLP-induced septic mice. To evaluate naringin’s effect on the survival of CLP-induced septic mice, mice were treated with either vehicle (DMSO, 0.1 ml per mouse, n = 5); naringin (200 mg/kg, intraperitoneal, n = 10); or naringin (200 mg/kg) + ZnPP (10 mg/kg, intraperitoneal, n = 10) 2 h prior to and 12 h after the operation. Survival was monitored every 24 h for up to 8 days. ^#^Significantly different from CLP-induced septic group.

**Fig 8:**
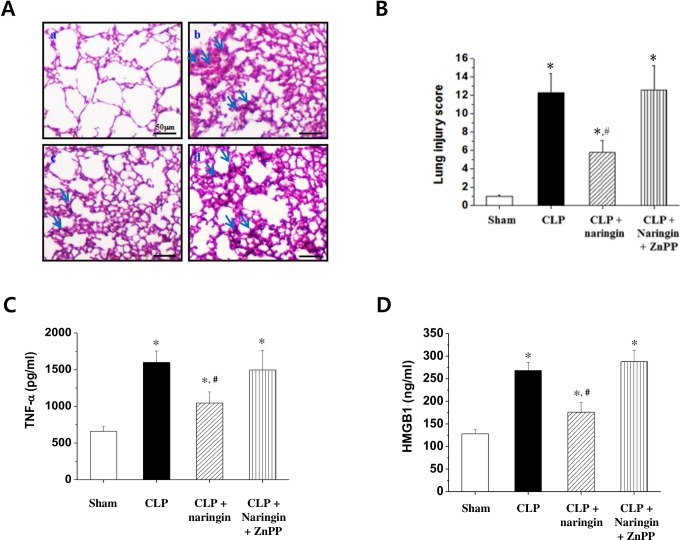
Naringin’s effect on histopathological changes in lung tissues and serum TNF-α and HMGB1 levels in CLP-induced septic BALB/c mice. The lungs from each experimental group were processed for histologic evaluation 1 day after CLP. Representative histologic changes in lung tissue obtained from mice belonging to each group can be seen and the arrows indicate the damaged area (hematoxylin and eosin staining; magnification, × 400); control (a), CLP (b), CLP + naringin (c), and CLP + naringin + ZnPP (d) (A). The lung injury was estimated with scores calculated in sections by assessing neutrophil infiltration, hemorrhage, necrosis, congestion, and edema and are shown on graph (B). Serum concentrations TNF-α (C) and HMGB1 (D) concentrations in each treatment group were measured by ELISA. Values are presented as means ± standard error (of the mean) (n = 4–6 in each group). *Significantly different from the sham group. ^#^Significantly different from the CLP-induced septic group.

Given that naringin exhibited protective effects in an endotoxin-induced mouse model of sepsis [16], we wondered if naringin would alleviate CLP-induced sepsis in mice via HO-1. As expected, administering naringin at 200 mg/kg significantly improved survival in a CLP-induced mouse model of sepsis; however, naringin’s protective effect was completely abolished by pre-treatment with the HO-1 inhibitor ZnPP (Fig 7). Lung damage is one of the leading causes of death in sepsis patients. Thus, we investigated whether HO-1 activity was related to naringin’s protective effect against lung injury in the CLP-induced model of sepsis. Histopathological features of lung injury were clearly seen in septic mice estimated with scores calculated by accessing neutrophil infiltration, hemorrhage, necrosis, congestion, and edema; naringin alleviated this damage; however, naringin’s protective effect was inhibited by ZnPP (Fig 8A and 8B). Naringin clearly reduced lung injury by increasing HO-1 expression in lung tissues. Given naringin’s inhibitory effect on inflammatory cytokines, we investigated naringin’s ability to attenuate CLP-induced TNF-α and HMGB1 expression in mice. Serum levels of the inflammatory cytokines TNF-α and HMGB1 were significantly increased 6 h after CLP induction. The CLP-induced increase in TNF-α and HMGB1 levels was suppressed by naringin; this inhibitory effect was abrogated by the HO-1 inhibitor ZnPP (Fig 8C and 8D). Overall, these results suggest that naringin can protect against CLP-induced death, lung injury, and increased TNF-α and HMGB1 expression, through the induction of HO-1 expression.

## Discussion

Natural products can modulate immune function by regulating various cytokines. The citrus flavanone naringin is an immune-regulatory natural product. Its therapeutic potential has been reported d for various diseases including atherosclerosis, cardiovascular disorders, and cancers [15]. Previous studies have shown that naringin protects against LPS-induced endotoxin shock in mice and RAW264.7 macrophages [16,18,24], and upregulates HO-1 expression via phosphatidylinositol 3-kinase/Akt, ERK1/2, protein kinase C-α, and NF-E2-related factor-2 in H9C2 myoblast cells [25]. However, the precise mechanism underlying naringin’s anti-septic action in macrophages remained unclear. Here, we show direct evidence that naringin contributes to reduced TNF-α and HMGB1 release by inducing HO-1 expression in LPS-stimulated macrophages.

In our in vivo and in vitro experiments in this study, naringin partially blocked the expression of HMGB1 and TNF- α in inflammatory condition. Only in Fig 1D, naringin completely reversed the LPS-induced increase of cellular concentration of HMGB1 and TNF- α. However, cellular amount of the secreted cytokines is not directly linked to the biological effects in inflammation, and total intracellular amount of each cytokine is determined by the balance of synthesis, degradation, and secretion. The secreted amount of cytokines in vitro system and serum level of cytokines represent the amount of functional cytokines in inflammation. Therefore, we suggest that naringin-induced pathway plays only partial, but significant role, in the prevention of LPS-mediated effects in vitro and CLP-medicated sepsis in vivo.

HMGB1 is a cytokine mediator of lethal systemic inflammatory conditions like sepsis [10,26], whereas HO-1 protects against sepsis-induced lung injury by blocking the release of HMGB1 [27,28]. Therefore, we assessed the signaling pathway(s) involved in naringin-induced HO-1 expression in macrophages. AMPK was recently shown to be a novel inducer of HO-1 expression in many cell types, including macrophages [29,30]. Moreover, previous studies have reported that crosstalk between the Nrf2 and AMPK signaling pathways is important for an alkaloid’s anti-inflammatory effect in LPS-stimulated macrophages [19], and that AMPK can mediate HO-1 expression via the downstream activation of p38 and Akt in J774 macrophages [31]. Here, we have provided evidence that AMPK and ACC (downstream AMPK target) phosphorylation stimulated HO-1 expression by silencing AMPK. Interestingly, AMPK silencing also reduced naringin-induced p38 MAPK and Nrf-2 activation. Indeed, many MAPK signaling pathways play a central role in inducing HO-1 expression [32,33]. We used specific MAPK inhibitors to show that p38 MAPK, but not JNK or ERK, mediated naringin-induced HO-1 expression, and that naringin activated p38 MAPK. Therefore, the results of the AMPK silencing experiments suggest a key role of AMPK in relaying naringin-induced stimulation of the signaling pathway to the downstream effectors p38, Nrf-2, and HO-1. Our findings indicate that HO-1 could be a target gene for AMPK.

Inducing HO-1 expression inhibits the expression of proinflammatory mediators like TNF-α through NF-κB inactivation [34]. We found that naringin’s suppression of NF-κB luciferase activity was reversed in the presence of a p38 MAPK inhibitor, AMPK silencing, and an HO-1 inhibitor. This finding suggests that naringin inhibits NF-κB luciferase activity, possibly by the induction of HO-1 expression via the AMPK and p38 MAPK signaling pathways. Thus, AMPK and p38 MAPK seem to play an important role in naringin-induced NF-κB inhibition in LPS-stimulated macrophages. However, the detailed molecular mechanism underlying naringin-induced AMPK activation remains to be elucidated.

The CLP model is the most representative mouse model of human sepsis [35]. Naringin protected mice from CLP-induced sepsis in an HO-1-dependent manner by reducing serum TNF-α and HMGB1 levels. Moreover, naringin administration (200 mg/kg) significantly reduced lung injury scores and protected against CLP-induced mortality. This was reversed by ZnPP treatment, suggesting that HO-1 activity plays a major role in naringin-mediated protection. Indeed, these results in animal models suggest naringin’s therapeutic potential in sepsis.

Various kinds of pro-inflammatory and anti-inflammatory cytokines are involved in sepsis pathology [36]. In this study, we only measured serum TNF-α levels (at 1 h) as the representative cytokine for the early stage of sepsis and HMGB1 levels (at 24 h) for the late stage. Neutrophil trafficking into inflamed sites is coordinated by various chemokines including CXCL1 [37]. However, CXCL1 expression was not significantly different in naringin-treated CLP mice (date not shown). Effects of naringin on other inflammatory cytokines and chemokines remain to be investigated in a future study.

In summary, naringin induces anti-inflammatory activity in sepsis by inducing HO-1 expression in macrophages through the AMPK, p38, and Nrf-2 signaling pathways. These results suggest that naringin is a potential therapeutic agent for sepsis.

## Abbreviations

ACC: acetyl-CoA carboxylase
AMPK: 5' adenosine monophosphate-activated protein kinase
ARE: antioxidant response element
CLP: cecal ligation and puncture
DMSO: dimethyl sulfoxide
ELISA: enzyme-linked immunosorbent assay
ERK: extracellular-signal-regulated kinase
HMGB1: high-mobility group box 1
HO-1: heme oxygenase 1
JNK: c-Jun N-terminal kinase
LPS: lipopolysaccharide
Nrf2: NF-E2-related factor 2
NF-κB: nuclear factor kappa-light-chain-enhancer of activated B cells
MAPK: mitogen-activated protein kinase
MTT: 3-(4,5- dimethylthiazol-2-yl)-2,5-diphenyltetrazolium bromide
RT-PCR: real-time polymerase chain reaction
SDS-PAGE: sodium dodecyl sulfate polyacrylamide gel electrophoresis
TNF-α: tumor necrosis factor-alpha
ZnPP: zinc protoporphyrin
